# Depression and Psychological Stress Among Health Workers in Remote Areas in Indonesia

**DOI:** 10.3389/fpubh.2022.743053

**Published:** 2022-04-27

**Authors:** Sri Idaiani, Lukman Waris

**Affiliations:** ^1^National Research and Innovation Agency, Jakarta, Indonesia; ^2^Department of Public Health, Universitas Falatehan, Banten, Indonesia

**Keywords:** health worker, remote area, depression, psychological stress, motivation

## Abstract

**Background:**

The Indonesian government launched the Nusantara Sehat program in 2015, under which teams of health workers were assigned to community health care centers in remote, border, and island areas for 2 years. The deployment to remote areas is likely to affect their psychological condition if they are not equipped with facilities and strong motivation. This study aimed to describe the psychological condition of health workers in remote areas in Indonesia, focusing on the proportion of the prevalence of depression and psychological stress.

**Materials and Methods:**

This cross-sectional study was conducted between April and December of 2018. Participants were 140 health workers placed in 26 community healthcare centers in 13 provinces. Interviews were conducted by enumerators using a questionnaire that included questions from the Mini International Neuropsychiatric Interview (MINI) and Self-Reporting Questionnaire-20 (SRQ-20).

**Results:**

Of the participants, 7.1% experienced depression and 10.0% experienced psychological stress. Motivation was related to psychological stress in participants with an odds ratio of 0,218 (95% confidence interval = 0.065–0.729, *p* = 0.013). Health workers with high motivation tend not to experience psychological stress compared to individuals with lower motivation.

**Conclusion:**

Health workers with high motivation experience relatively low levels of psychological stress. To overcome stress, high motivation is needed to control psychological risk factors before and during placement.

## Introduction

Living in a remote area, far from family and friends, with a lack of facilities is a stressor for health workers (1). The most common psychological disorder among workers is burnout. Burnout generally occurs in workers in humanitarian and social fields. There are three aspects of burnout: emotional exhaustion characterized by anxiety and depression, depersonalization, and personal achievement (2). Emotional exhaustion has been found to have the highest correlation with workers' mental health (3).

Various factors, both internal and external, influence the psychosocial condition of health workers. Internal factors include workers' health, social and family networks, health-related behavior, and motivation, while work environment, residence, workload, income, supervision, guidance, national politics, national policies, income inequality, and employment conditions constitute external factors (4).

From the preceding description, it is clear that emotional fatigue often occurs in workers in the form of anxiety and depression, and also, in a milder form, general stress or psychological stress. Anxiety is more commonly characterized by autonomic symptoms, and depression by low mood. Unresolved psychological stress is likely to develop into more specific disorders, such as anxiety, depression, and other psychological conditions. In Indonesia, according to the National Health Survey (NHS), the prevalence of depression in the general population aged 15 years is 6.1%, while in the general population who have a job, it varies from 2.4 to 6.9%. Additionally, the prevalence of psychological stress in people over the age of 15 years is 9.8%, while among those who have a job is 3.9–10.8% (5).

Stress and depression, or other mental disorders, are known to decrease productivity; although many factors, such as motivation, affect them. Motivation is influenced by rewards, finances, and reinforcements, which is also related to other problems, such as workload and family factors (6). Decreased productivity causes losses for providers and, in the case of health workers, the government loses providers should healthcare workers decrease productivity. The government spends a considerable amount of money to place health workers and have to ensure that they work efficiently.

In 2015, the government of Indonesia launched the Team Based Nusantara Sehat (TBNS) program to meet health service needs. According to the Minister of Health Regulation Number 16 of 2017, each team consists of at least five types of health professionals, namely, general practitioners, dentists, public health workers, sanitarians, nutritionists, laboratory analysts, pharmacists, midwives, and nurses. The teams are placed for two years in community health care centers (CHCs) in remote areas far from the district capital that lack basic infrastructure. Remote areas, in this context, are determined by the Ministry of Village, Development of Disadvantaged Regions, and Transmigration by considering the economic factors, human resources, infrastructure accessibility, and other regional characteristics.

In general, the health workers deployed to remote areas are fresh graduates with a health education background. The maximum age limit is 30 years for general practitioners and 25 years for other health workers. The health workers are expected to smoothly and successfully pass the two-year deployment period. Those who fail to carry out their duties receive sanctions, making it difficult for them to participate in other health programs.

The TBNS personnel are placed in remote areas. Therefore, the government has to ensure that the deployed team is working optimally and productively. Considering this, the evaluation of both the physical and mental conditions of the team placed in remote areas is necessary. This paper will focus on the mental health of the team because poor psychological conditions lead to burnout and low productivity (3, 7). In addition, until recently, the mental condition of health workers in remote areas has never been assessed. Therefore, it is necessary to assess the mental status of health workers in remote areas. With this mind, the Ministry of Health, in 2018, approved a study to assess the psychological condition of health workers in remote areas in Indonesia. The purpose of this study was to describe the psychological conditions (depression and psychological stress) of health workers placed at CHCs in remote areas between 2015 and 2017.

## Materials and Methods

### Study Population

The study population was comprised with teams of health workers placed at 26 CHCs in remote areas across 13 provinces between 2015 and 2017 (waves 6 and 8). The CHCs were selected by random sampling from among 39 CHCs in 28 provinces. The health workers were selected by purposive sampling. We did not include health workers from previous waves as they had already completed the program. “Batch” or “wave” is the period when the team is deployed to the location. When this study was conducted, there were two waves or batches in the location. Wave 6 had been on location longer than wave 8. Deployment is considered a proxy for work experience.

As a subject criterion, all TBNS members who were in the selected location were eligible as participants. Members who were not present at the time the research team visited the site or were unwilling to participate in the study were excluded.

Minimum sample size was estimated using the formula *n* = *N* ^*^ *X*/(*X* + *N* – 1) where *X* = $Z_{\alpha /2}^{2}$ ^*^ *p* ^*^ (1 – *p*) / MOE^2^ (8), where *p* = 0.108 (from *p* which produces the most n, namely, p of psychological stress), *N* = 210 (prediction of total number of TNBS personnel with assumption 1 team consisted of 6 person from 35 CHCs), confidence level of 95%, α is 0.05 and *Z*_α/2_ = 1.96 thus $Z_{\alpha /2}^{2}$ = 3.8416), and MOE is the margin of error = 2%. Based on this formula, the minimum required sample size was 88.

### Measurements

The interview questionnaire collected demographic data and included items on motivation, depression, and psychological stress. Depression was measured using the depression questionnaire (9–11) from version 6 of the Mini International Neuropsychiatric Interview (MINI). The MINI is an interview-based diagnostic tool which assesses depression in the past 2 weeks or over the lifetime. It consists of three screening questions and seven main questions. The data from the tool are inputted into an algorithm which produces an output assessing whether a person is depressed or not. In Indonesia, the validity and reliability of the tool have been measured with good results (12, 13). Psychological stress was assessed using 20 questions from the Self-Reporting Questionnaire (SRQ) (14). Respondents were categorized as depressed if they met the requirements according to the MINI questionnaire, whereas those who answered “yes” to at least 6 of the 20 SRQ questions suffered from psychological stress, a condition characterized by symptoms of anxiety and depression. The cut-off point of 6 is applied to all characteristics of the participants, both men and women, according to the results of validity tests that have been previously carried out and have been used in routine surveys in Indonesia (15).

Motivation was assessed using a motivation questionnaire that was originally developed for workers in rural areas in Zambia (16). It consists of 23 questions about general motivation, job satisfaction, intrinsic job satisfaction, burnout, organization commitment, contentiousness, and timeliness using a Likert-type scale. Based on the assessment method, motivation was divided into three categories: low (score < 25), medium (score = 25–74), and high (score ≥ 75). There is no information regarding validation of this questionnaire in Indonesia; however, it has been adapted and translated into Indonesian and has been used in the 2017 National Health Worker Research (17). The data collectors (enumerators) read all questions to the respondents. The enumerators received training on data collection procedures and interviewing techniques related to the questionnaire material.

## Data Analysis

STATAⓇ version 14 used spmap command to display the location of the selected CHCs. IBMⓇSPSSⓇ version 24 was used to undertake a chi square test of independence to establish initial bivariate relationships between variables (gender, wave, type of health worker, motivation). We included variables in subsequent multivariate analyses if they were significant at p < 0.25. Included variables were simultaneously entered into two logistic regression equations – one using the outcome variable of depression and the other psychological distress. An association between predictor and outcome variables were assumed at p < 0.05.

## Results

**Figure 1:**
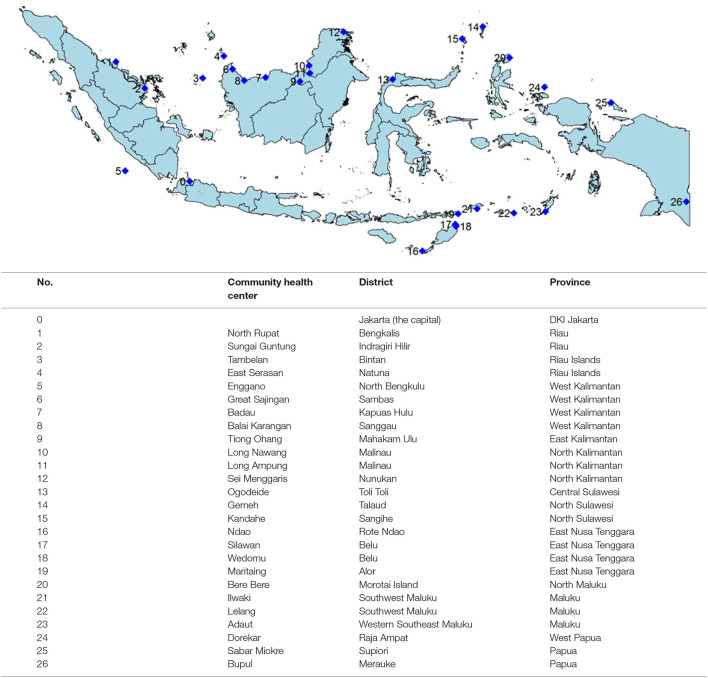
Location of selected community health centers.

Figure 1 shows that most community health centers (CHCs) are located in the outer border areas and islands. There were no selected CHCs in Java and Bali.

Table 1 details the distribution of community health centers (CHCs) and respondents by province. There were 140 respondents from 26 CHCs in 13 provinces.

**Table 1:** Number of community health centers and respondents by province.

**Province**	***n* CHC**	***n* Respondents**	**% Respondents**
Riau	2	10	7.1
Bengkulu	1	6	4.3
Riau Islands	2	11	7.9
West Kalimantan	3	17	2.1
East Kalimantan	1	5	3.6
North Kalimantan	3	18	2.9
North Sulawesi	1	6	4.3
Central Sulawesi	1	6	4.3
East Nusa Tenggara	4	22	15.7
Maluku	4	17	12.1
North Maluku	1	5	3.6
West Papua	1	6	4.3
Papua	2	11	7.9
Total	26	140	100

The proportion of health workers who experienced depression was 7.1%, while 10.0% experienced psychological stress (Table 2). There were no respondents with low motivation. Therefore, motivation is classified only as medium or high.

**Table 2:** Factors related to depression and psychological stress.

	**Depression**				*p* ^*^	**Psychological stress**				*p* ^*^
	**Yes**		**No**			**Yes**		**No**		
	***n***	**%**	***n***	**%**		***n***	**%**	***n***	**%**	
**Gender**										
Male	2	20.0	47	36.2	0.492	2	14.3	47	37.3	0.138
Female	8	80.0	83	63.8		12	85.7	79	62.7	
**Wave**										
6	1	10.0	62	47.7	0.023	3	21.4	60	47.6	0.089
8	9	90.0	68	52.3		11	78.6	66	52.4	
**Type of health worker**										
Doctor (general practitioner), dentist, midwife, nurse	2	20.0	51	39.2	0.319	3	21.4	50	38.7	0.296
Public health worker, sanitarian, nutritionist, laboratory analyst, pharmacist	8	80.0	79	60.8		11	78.6	76	60.3	
**Motivation**										
Medium	9	90.0	127	97.7	0.259	12	85.7	124	98.4	0.050
High	1	10.0	3	2.3		2	14.3	2	1.6	
Total	10	7.1	130	92.9		14	10.0	126	90.0	

* *Fisher's exact test*.

Wave of participation and motivation had a significant relationship with depression (*p* < 0.25). Psychological stress, wave and motivation, and gender, had a significant relationship (*p* < 0.25). Health workers in wave 6 were placed at CHCs ~6 months before those in wave 8. The variables of interest were subjected to multivariate analysis and the results are displayed in Tables 3, 4.

**Table 3:** Results of multivariate analysis of factors related to depression.

	**Depression**				
	**OR**	**SE**	***Z***	**95% CI**	***p***
**Wave**					
6	Ref				
8	0.132	0.141	−1.89	0.016–1.078	0.059
**Motivation**					
Medium	Ref				
High	0.342	0.234	−1.56	0.089–1.312	0.118

*OR, odds ratio; CI, confidence interval*.

**Table 4:** Results of multivariate analysis of factors related to psychological stress.

	**Psychological stress**				
	**OR**	**SE**	***Z***	**95% CI**	***p***
**Gender**					
Male	Ref				
Female	4.484	3.675	1.83	0.045–1.234	0.067
**Wave**					
6	Ref				
8	0.287	0.202	−1.77	0.087–1.302	0.076
**Motivation**					
Medium	Ref				
High	0.218	0.134	−2.47	0.065–0.729	0.013

*OR, odds ratio; CI, confidence interval*.

Table 3 shows that more recent work periods (wave 8) have almost no effect on depression (confidence interval is 0.016–1.078). Motivation is also not related to depression (*p* > 0.05). Table 4 shows a significant relationship between motivation and psychological stress. That is, people with high motivation have lower risk of psychological stress compared to those with medium motivation (*p* = 0.013). Both gender and wave of participation did not show any relationship with psychological stress (confidence interval for gender is 0.045–1.234 and wave is 0.087–1.302, *p* > 0.05).

## Discussion

The proportion of remote health workers who experienced depression was 7.1%. This number was higher than the national rate of 6.1% for population aged ≥15 years based on the NHS conducted in 2018 (14). The NHS assessed depression using the same tool as this study, that is, the depression questionnaire from MINI version 6. The prevalence of psychological stress among remote health workers was 10%, which was also similar to NHS 2018 (9.8% in population aged ≥15 years).

The prevalence of depression and psychological stress varies depending on the type of work. According to NHS 2018, in general, the prevalence of depression (2.4–6.9%) and psychological stress (3.9–10.8%) in workers is lower than the national rate. Comparing the results of this study with those of other studies is difficult because previous studies assessed depression in health workers in disaster areas (18, 19) and not in remote areas.

Although gender was not associated with depression and psychological stress, the prevalence of depression was higher in women than in men. This is consistent with the results of studies on mental health in general and NHS 2018. As for stress in the workplace, men tend to ignore the symptoms of depression (20). Another study states that depression and anxiety at the workplace are associated with musculoskeletal disorders (21). Furthermore, victims of workplace violence in health care settings are generally women, widows, and youngsters who are at high risk of psychiatric morbidity (19).

Health workers who were often absent from duty appeared to have more depression and stress, although there were no statistically significant differences. This is similar to the results of previous studies that absence at work is related to stress, fatigue, and burnout (3). In a study of ethnic Latin workers, most cases of depression, though moderate in degree, occurred during the first year of work. The questionnaire used in this study did not categorize the degree of depression (22).

In this study, the type of profession did not have an association with depression or psychological stress. Because of the small number of respondents, some professionals were placed into two groups. In general, health workers have a similar risk of experiencing stress as other professionals. Moreover, as doctors and dentists are more experienced than other health workers, they are better able to cope with stress. Doctors and dentists have the possibility of starting an independent practice after the completion of the health program, whereas other health workers are dependent on employment opportunities at institutions or healthcare facilities.

Furthermore, work motivation only related to psychological stress in this study. Work motivation is influenced by many factors such as leadership, job satisfaction, income, social support, and work skills (23–26). Therefore, living in remote areas is not the only factor influencing the retention of health workers.

Other factors that affect health workers in remote areas include health system, financial and socio-economic conditions, job satisfaction, retention, and turnover (27–29). Studies on health workers in remote areas have discussed turnover intention as a cause of burnout and stress (28, 30). As for stress at work, studies have generally focused on informal or part-time workers. Part-time male workers tend not to complain significantly (31). Stress can have both long- and short-term effects. Long-term stress affects the retirement period, while short-term stress causes a lack of productivity, which ultimately affects the quality of health services (20, 32).

It is thus important to deal with stress and depression at work so as not to affect the quality of service. The ways of dealing with stress vary depending on age and other factors. Providing clear work instructions and conducting risk assessments is one way to prevent stress in the workforce (33–35).

This study has several limitations. First, a number of important determinants of depression and psychological stress were not examined, such as job satisfaction, career pathway, income, social support, turnover, availability of medicines, skills, safe and supportive environment, and access to health facilities (28, 36). Second, the cross-sectional design limits the inference and direction of causality. The strength of this study is that it was conducted on a hard-to-reach population, given the geography of Indonesia. It focuses on a population that had not been previously studied in the country. Second, it utilized standardized instruments for the diagnosis of depression and the measurement of psychological stress.

## Conclusion

The prevalence of depression and psychological stress among health workers in remote areas of Indonesia is slightly higher than in the general adult population of Indonesia. This number is also higher compared with other types of workers in Indonesia. Motivation is one of the factors associated with psychological stress. In this study, there was no association between respondents' working experience and gender with both depression and psychological stress.

These results should be further interogated with studies specifically assessing the psychological conditions of health workers in remote areas using a larger sample size and varying working periods or wave placements. In terms of work place policy, the Health Office should conduct periodic supervision to improve motivation and skills and ensure the welfare of remote health workers throughout Indonesia. Stress risk control is required both before and during placement, such as stress management, work training, and supervision.

## Data Availability Statement

The raw data supporting the conclusions of this article will be made available by the authors, without undue reservation.

## Ethics Statement

The study protocol was reviewed and approved by the Scientific Committee and Health Research Ethics Commission of the National Institute of Health Research and Development in Indonesia (number: LB.02.01/2/KE.116/2018). All procedures were performed in accordance with the Helsinki Declaration. Written informed consent was obtained from all the participants prior to the interviews.

## Author Contributions

SI participated in manuscript writing and data analysis. LW led the project and made revisions to the manuscript. All authors read and approved the final manuscript.

## Funding

This study was supported by the Indonesian Ministry of Health. The fund was directed to cover the travel and accommodation of researchers from Jakarta to study locations.

## Conflict of Interest

The authors declare that the research was conducted in the absence of any commercial or financial relationships that could be construed as a potential conflict of interest.

## Publisher's Note

All claims expressed in this article are solely those of the authors and do not necessarily represent those of their affiliated organizations, or those of the publisher, the editors and the reviewers. Any product that may be evaluated in this article, or claim that may be made by its manufacturer, is not guaranteed or endorsed by the publisher.
